# Linearized texture of three-dimensional extracellular matrix is mandatory for bladder cancer cell invasion

**DOI:** 10.1038/srep36128

**Published:** 2016-10-25

**Authors:** Massimo Alfano, Manuela Nebuloni, Raffaele Allevi, Pietro Zerbi, Erika Longhi, Roberta Lucianò, Irene Locatelli, Angela Pecoraro, Marco Indrieri, Chantal Speziali, Claudio Doglioni, Paolo Milani, Francesco Montorsi, Andrea Salonia

**Affiliations:** 1 Division of Experimental Oncology/Unit of Urology, URI, IRCCS Ospedale San Raffaele, Milan, Italy; 2 Pathology Unit, Department of Biomedical and Clinical Sciences, L. Sacco Hospital, Università degli Studi di Milano, Milan, Italy; 3 Centro di Microscopia Elettronica per lo studio delle Nanotecnologie Applicate alla medicina “C.M.E.N.A.”, Department of Biomedical and Clinical Sciences, L. Sacco Hospital, Università degli Studi di Milano, Milan, Italy; 4 Pathology Unit, IRCCS Ospedale San Raffaele, Milan, Italy; 5 Fondazione Filarete, viale Ortles 22/4, 21033, Milan, Italy; 6 Università Vita-Salute San Raffaele, Milan, Italy; 7 Interdisciplinary Centre for Nanostructured Materials and Interfaces (CIMaINa) and Dept. of Physics, Università degli Studi di Milano, Milan, Italy

## Abstract

In the fields of biomaterials and tissue engineering simulating the native microenvironment is of utmost importance. As a major component of the microenvironment, the extracellular matrix (ECM) contributes to tissue homeostasis, whereas modifications of native features are associated with pathological conditions. Furthermore, three-dimensional (3D) geometry is an important feature of synthetic scaffolds favoring cell stemness, maintenance and differentiation. We analyzed the 3D structure, geometrical measurements and anisotropy of the ECM isolated from (i) human bladder mucosa (basal lamina and lamina propria) and muscularis propria; and, (ii) bladder carcinoma (BC). Next, binding and invasion of bladder metastatic cell line was observed on synthetic scaffold recapitulating anisotropy of tumoral ECM, but not on scaffold with disorganized texture typical of non-neoplastic lamina propria. This study provided information regarding the ultrastructure and geometry of healthy human bladder and BC ECMs. Likewise, using synthetic scaffolds we identified linearization of the texture as a mandatory feature for BC cell invasion. Integrating microstructure and geometry with biochemical and mechanical factors could support the development of an innovative synthetic bladder substitute or a tumoral scaffold predictive of chemotherapy outcomes.

A comprehensive examination of the 3D-structure and geometry of the extracellular matrix (ECM) of non-tumoral human bladder and paired human urothelial bladder cancer (UBC) specimens have not yet been undertaken. Given the proven critical role of ECM in determining cell behavior, organ functioning, and tumor progression^1^,^2^,^3^,^4^, a through characterization of the healthy and tumoral human bladder ECM would pave the way to (i) assessing pathways leading to modification of bladder ECM during tumor progression and invasion; (ii) modelling a 3D synthetic bladder wall able to create an environment more similar to the *in vivo* microenvironmental niche conditions to test innovative therapies; and, (iii) for promoting bladder regenerative medicine in patients eventually needing bladder augmentation. In this context, we sought to assess the ultrastructure and geometry of the healthy human bladder ECM; moreover, specimens from muscle invasive bladder cancer (MIBC) were used to assess modifications of the ECM that occurred during tissue invasion. Therefore, we provide novel findings regarding an ultrastructural analysis of the ECM from the basal lamina, lamina propria, and muscularis propria layers of the healthy bladder, comparing the features of non-tumoral ECMs with that of MIBC-derived ECM.

Either partial or complete urinary bladder replacement may be indicated in individuals with congenital or acquired diseases (eg, congenital bladder extrophy and spina bifida, spinal cord injury and MIBC) with indications for orthotopic bladder reconstruction^5^,^6^. Currently, the treatment of choice in these cases is surgical enlargement of the bladder using intestinal tissue. Enterocystoplasty is potentially associated with a number of sequalae such as metabolic disturbance (iperchloremic acidosis), increased mucus production, urinary stones formation, urinary tract infections and even secondary-developed malignant diseases^7^. In an attempt to overcome these limitations, over the last decades naturally-derived and synthetic scaffolds have been engineered with the specific aim to provide alternatives for either repairing or replacing the bladder^8^.

Being an hollow organ composed of different tissue layers, bladder substitutes must provide support for (i) the high density of urothelial and smooth muscle cells (eg, serving as a barrier between luminal contents and the body cavity, while preventing premature collapse of the hollow organ itself); and, (ii) the formation of properly-oriented muscle tissue, innervation and vascularization. Unfortunately both naturally-derived and synthetic scaffolds, either transplanted alone or following the seeding of autologous cells, have shown limited success, mainly due to the lack of proper innervation and blood supply, thus leading to graft shrinkage and a lack of functionality^8^.

The extracellular environment is made of ECM composed of structural (core-matrisome) and matrisome-associated components^9^. ECM proteins and proteoglycans interact within a network but also participate in supra-molecular assemblies where their biological properties are modified. Conversely, matrisome-associated components (eg, cytokines, growth factors, matrix metallo-proteases) bind to the glycosylated components of the ECM, thus localizing their mechanism of action in a specific compartment. Moreover, intrinsic domains of stromal proteins have growth factor-like structures which act as ligands for canonical growth factor receptors. Therefore, ECM has been described as an organized solid-phase ensemble of ligands^10^. In addition to the biochemical signalling provided to cells by ECM proteins, stiffness, topography and geometry of the extracellular space also modulate cell behaviour and phenotype^1^,^2^,^3^. Recently, three-dimensional geometry has been reported as an important feature of synthetic scaffolds favoring cell stemness, maintenance and differentiation, nerve regeneration and vascularization^11^,^12^,^13^,^14^. Further evidence of the role of matrix geometry is the fact that a depletion of all integrins does not impede the movement of dendritic cells in 3D matrices^15^, whereas matrix geometry and the resulting cell confinement were the main parameters regulating cell phenotype and cell behavior^16^,^17^,^18^.

Modifications of the ECM emerged to be of major importance in terms of tumor progression, both at the primary site^19^ and in metastatic niches^20^. Recent evidence has suggested that tumor-related extracellular environment may play a leading role, and it is not just a supporting actor over the initiation of the tumor; indeed, the dysregulation of the composition^4^ and stiffness^21^ of the ECM are associated with both the lack of asymmetric cellular division and of the differentiation of stem cells, and with the epithelial-mesenchymal transition of cancer stem cells as well, thus regulating tissue homeostasis and sustaining the onset and progression of cancer^4^,^19^. In agreement with these latter observations, we recently reported that an increased organization of ECM fibrils, but not biochemical composition, of the perilesional area of the colorectal carcinoma^22^ is associated with proliferation and infiltration of metastatic cancer cells^23^.

Here we established ultrastructural features of tissue layers ECM of the human bladder, detailing those modifications occurring with MIBCs.

Next, feasibility study was performed to evaluate if the ultrastructural geometry of MIBC stroma was sufficient to allow for adhesion and infiltration of bladder metastatic cells, using a synthetic matrix recapitulating the texture of MIBC stroma. Since the MIBC stroma has a sub-fibrous morphology, we have produced the synthetic matrix using the electrospinning technique, widely used in regenerative medicine and in bladder tissue engineering^24^,^25^,^26^,^27^, and polycaprolactone (PCL) as a polymer model^25^. Electrospinning uses charge separation to produce micro- to nanoscale non-woven fibrous mats from a polymeric solution. It is accomplished by inducing a large electric potential (10 to 30 kV) in a polymer solution (synthetic or natural) and separating that polymer from an oppositely charged target (collector). This charge separation creates a static electric field that drags the polymer fibers to the collector. During the time of flight of fibers to the collector the solvent of the solution evaporates and dry fibers are then collected in the form of non-woven mats on the collector. Varying the type of collector and the polymer solution concentration and electrospinning parameters it is also possible to obtain random, aligned or twisted fibers. The base polymer used in the herein feasibility study, the PCL, has been chosen for its wide use in electrospun tissue engineering^28^, since it allows to control the pore size of membrane by changing the fibers density in unit area^29^. PCL is also biocompatible, biodegradable and, in the form of electrospun membranes, it has mechanical properties (Young Modulus of 3.8 ± 0.8 MPa)^30^ adequate to support the adhesion and proliferation of cells derived from human bladder^31^.

We have produced 2 types of scaffolds, changing the collector type (rotating drum and grid collector) but without varying the polymer. Each scaffold has been characterized for superficial morphological and geometrical characteristics, and tested for the adhesion and infiltration of bladder cancer cells T24. The different production set-up leaded to different scaffold features, such as geometrical pattern characterized by disorganized and organized degree of fibril organization, with the latter recapitulating anisotropy of MIBC stroma and allowing cell invasion.

This synthetic scaffold recapitulating morphologic features of the bladder environment needed for tissue invasion might aid in the identification of new therapeutic targets and drugs for controlling the outcome of urothelial bladder cancer. In general, this synthetic scaffold might be a successful and more predictable future 3D platform^32^ for the pre-clinical research and for better understanding the process of bladder cancer development.

## Results

### Patient characteristics

According to the increased incidence of UBC in male vs. female individuals, paired non-tumoral and tumoral bladder areas were collected from specimens of 10 male individuals submitted to radical cystectomy for MIBC. Inclusion criteria only considered UBCs, while excluding squamous and sarcomatoid histotypes (Table 1).

Non-neoplastic tissue was obtained from the anterior side of the bladder, which emerged as the most distant area from the tumor lesion in every individual (Table 1). Furthermore, the choice of the anterior side of the bladder allowed to avoid the bias caused by the heterogeneity of the bladder tissues, that is usually limited to the areas of the bladder neck and the trigone, where the thickness of the lamina propria is thinner as compared with that in the dome and in the left/right/anterior and posterior wall of bladder due to an increased size of the muscularis propria^33^.

**Figure 1:**
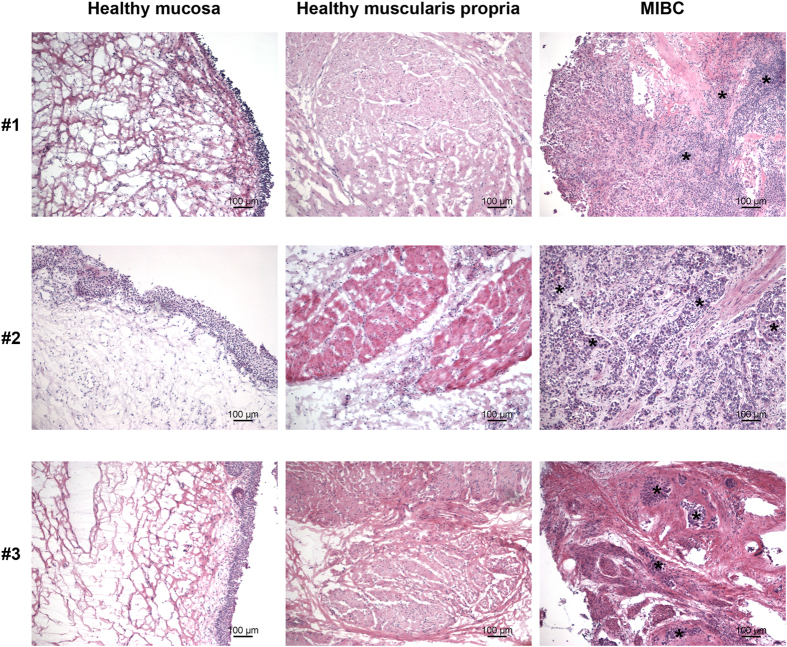
Histology of bladder mucosa, muscularis propria and MIBC. Paired mucosa and muscularis propria were selected from the non-neoplastic tissue vs MIBC from cystectomies and evaluated by hematoxylin-eosin staining. Three donors out of the 10 tested are shown. Asterisks are representative of areas infiltrated by tumor cells.

Validation of both non-neoplastic and neoplastic areas was performed by histological analysis at light microscopy. Bladder layers were separated into the mucosa (urothelium and lamina propria) and the muscle layer. All non-neoplastic bladder areas were free of epithelial dysplasia and/or inflammatory infiltrate; conversely, MIBC areas showed cancer cells infiltrating both the sub-epithelial stroma and the muscle layer (Fig. 1).

### ECMs from non-neoplastic bladder tissue and MIBC maintain the architecture ascribed to the native tissue

**Figure 2:**
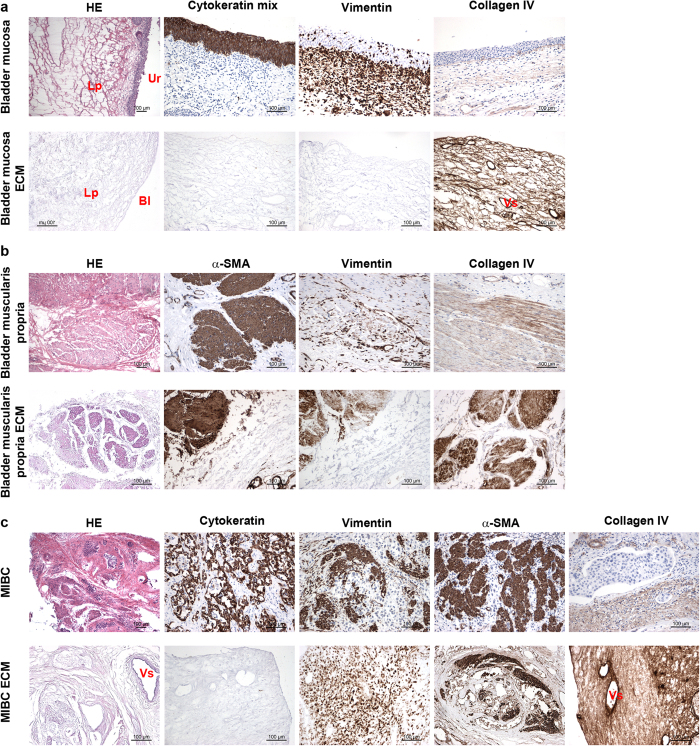
Histology and immunohistochemistry analysis of ECMs from non-neoplastic bladder mucosa and muscularis propria and MIBC. ECM from bladder mucosa (**a**) muscularis propria (**b**) and MIBC (**c**) were evaluated for the absence/presence of cells and stromal makers. Presence and absence of cells and nuclei in the bladder layers was evaluated by means of hematoxylin-eosin staining. Presence and absence of epithelial, stromal and smooth muscle cells in bladder layers and the relative ECMs was evaluated by means of the expression of cytokeratin, vimentin and α-smooth muscle actin (α-SMA), respectively. Expression of collagen IV was used as marker of ECM. All tested bladders showed similar result, and representative pictures from one donor are shown. Paired tissue and ECM are shown from one representative bladder. HE; hematoxylin-eosin, Ur; urothelium, Lp; lamina propria, Bl; Basal lamina, Vs; Vessel.

ECMs from human bladder mucosa (Fig. 2a), bladder muscularis propria (Fig. 2b) and MIBC (Fig. 2c) were isolated according to our previously published protocol validated on the human colon^23^. The hematoxylin-eosin stained sections of the non-neoplastic urinary bladder showed the different cells layers: (i) mucosa, formed by transitional epithelium and lamina propria, and (ii) muscolaris propria, known as detrusor muscle, formed by three layers of smooth muscle. MIBC contained a disorganized tissue structure with neoplastic cells infiltrating the muscle layer. Hematoxylin-eosin staining of mucosa, muscolaris and MIBC ECM revealed that the architecture ascribed to the native tissue was maintained, while lacking cells and nuclei.

Immunohistochemistry was used to identify cellular and stromal components and to validate ECM procedure. Absence of cytokeratin staining revealed lack of epithelial cells in the ECM from the mucosa and MIBC. Vimentin staining showed the removal of stromal cells in the ECM from mucosa, whereas positive labeling remained in the ECM from muscularis propria and MIBC. In agreement with this information, also the staining with α-smooth muscle actin revealed maintenance of cytoskeletal elements of stromal origin, likely binding the surrounding matrix. The stromal protein type IV collagen was maintained in the ECM with the usual distribution, as evident by its expression surrounding capillary structures in the lamina propria and vessels in MIBC-ECM.

### ECMs from bladder lamina propria, muscularis propria and MIBC have 3D ultrastructural differences

**Figure 3:**
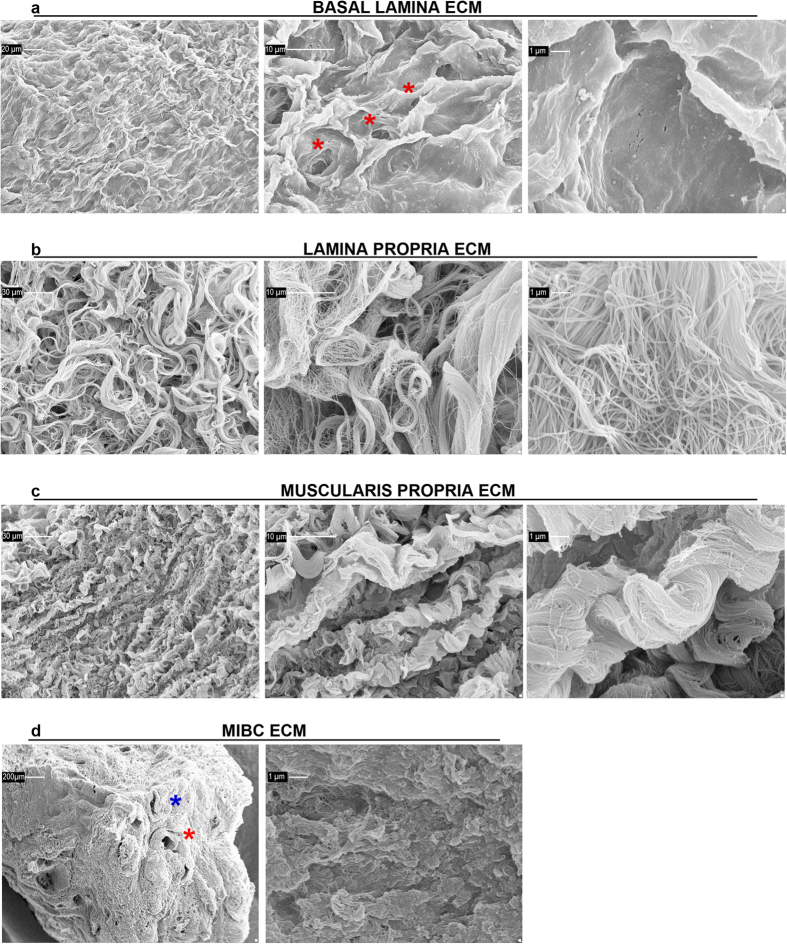
Ultrastructure of ECM isolated from human bladder mucosa, muscularis propria and MIBC. Ultrastructure of ECM derived from bladder mucosa, muscularis propria and MIBC were analyzed by scanning electron microscopy. Ultrastructure of basal lamina ECM (**a**) lamina propria ECM (**b**) and muscularis propria ECM (**c**) are reported at magnification of 1000x, 5010x and 20030x (with scale bars reported): red asterisks in panel A indicate capillaries. The ultrastructure of the MIBC ECM was analyzed by scanning electron microscopy at 100x and 20030x magnifications (scale bars reported) (**d**) red and blue asterisk indicate representative artery and vein, respectively. Representative pictures of ECM ultrastructure from tissues obtained from one cystectomy are shown.

The ultrastructural evaluation performed via scanning electron microscopy analysis of the ECM from non-tumoral bladder mucosa revealed a different morphology of the two tissue layers. The healthy basal lamina ECM showed a flat surface characterized by regular “footprint-like” depressions with a compact base; in some of the footprint-like” depressions round-shaped empty spaces of 6 ± 0.5 μm (28 counts from the 10 tested mucosae-derived ECMs) representative of empty capillaries were present (Fig. 3a), as observed with type IV collagen staining. The ECM from the healthy lamina propria showed a wavy pattern constituted of well-separated but intersecting fibrils forming a random network (Fig. 3b).

The ultrastructure of ECM from the bladder muscularis propria appeared to have a regular morphology of crests and clefts and a well-oriented direction of fibers consisting of parallel fibrils organized in intertwisted bundles (Fig. 3c).

The ultrastructure of the MIBC-derived ECM was characterized by many empty “holes” remaining after the removal of cells, which were ascribed to veins and arteries (Fig. 3d), based on shape and diameter (ranging between 25 and 235 μm, in agreement with previously reported diameters of veins and arteries in the healthy bladder^34^; 61 counts from the 10 tested MIBC-derived ECM). The ECM from MIBC was also characterized by a dense and compacted layer (Fig. 3d).

### MIBC ECM is characterized by increased linearized texture

**Figure 4:**
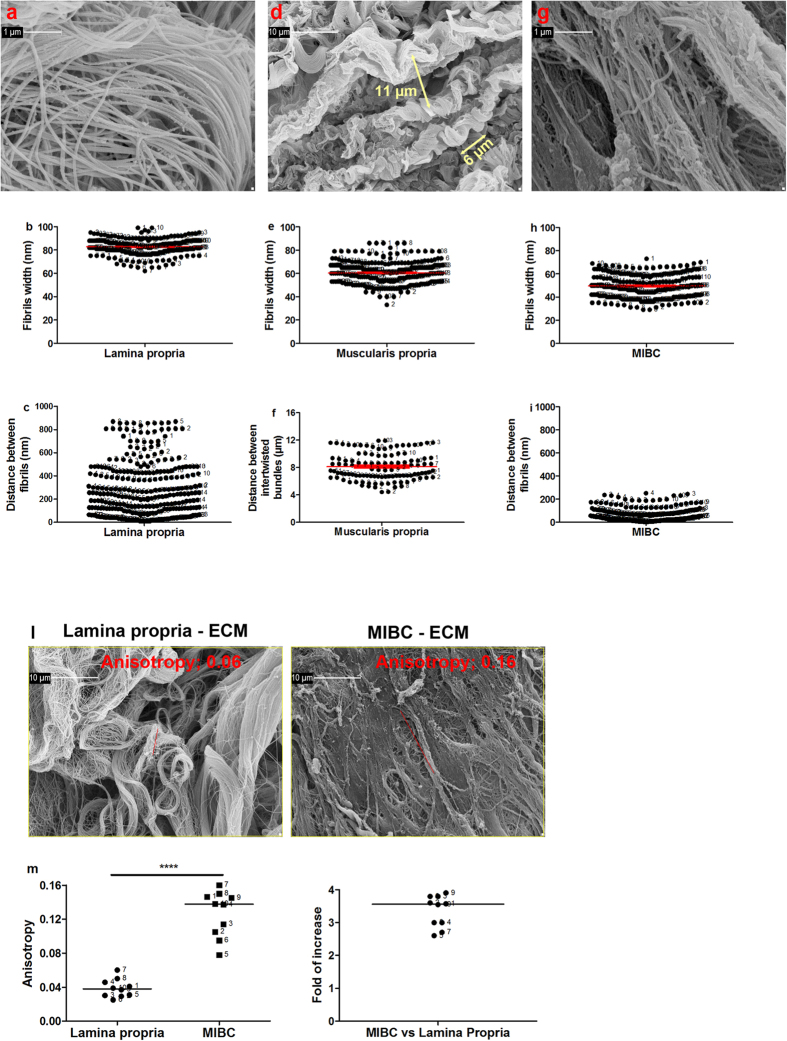
Texture of bladder ECMs and MIBC ECM. Geometry of the ECM ascribed to bladder lamina propria (**a**) muscularis propria (**d**) and MIBC (**g**) was established using SEM pictures (40060x magnification in panels (a) and (g), 5010x magnification in panel (d) Double headed arrows in panel d are representative of how the distance between bundles and the distance among three twists was measured. Numbers within the graph represent multiple measurements of tissue-derived ECM from the 10 patients. Red bars in panels b,e,h and f represent mean ± standard error mean. The degree of alignment of fibrils was estimated on SEM micrographs of ECM from non-neoplastic lamina propria and MIBC (**l**). Anisotropy was estimated on the region of interest inside the colored box, and the output is represented by the red line, the angle of which represents the average orientation and the length of which is proportional to the degree of alignment. Anisotropry of ECM from pair-wise tissues and fold of increase of tumoral stroma vs non-neoplastic stroma are reported (**m**). Horizontal bars indicate median value, numbers within the graphs indicate anonymized patients, and asterisks show statistical significance evaluated by means of 2 tailed paired T-test.

Width of fibrils in the lamina propria was 83 ± 0.4 nm (mean ± SEM) with an inter-fibrils distance ranging between 10 and 870 nm (Fig. 4a–c). Fibrils width in the muscularis propria was 60 ± 0.6 nm, with fibrils appearing to be organized in intertwisted bundles, with inter-bundles distances of 8 ± 0.2 μm (Fig. 4d–f). On average, the bundles completed a twist in 4.7 μm (6.7 μm was the median distance among three twists, represented in Fig. 4d).

The dense and compact layer of MIBC ECM was composed by fibrils with a width of 50 ± 0.6 nm, therefore thinner than the width of those present in the non-tumoral counterparts of both the lamina propria and the muscularis propria (p < 0.0001, Krustal Wallis test); likewise, MIBC ECM presented fibrils more condensed together (with an inter-fibrils distance in ranging between 2 and 250 nm) (Fig. 4g–i). In agreement with these findings, the texture of MIBC ECM was thus characterized by an increased degree of organization of fibrils (Fig. 4l), with a fold of increase vs non-neoplastic ECM (Fig. 4m), similar to that previously reported in colorectal^22^ and ovarian cancers^35^.

### Anisotropic architecture of PCL scaffold influences binding and invasion of metastatic bladder cancer cell line T24

Next, we attempted to design synthetic model recapitulating the features of tissue-derived ECM sustaining cell invasion, focusing our model on the role of the texture of fibrillar organization. To this regard, 2 scaffolds of PCL nanofibers were designed.

**Figure 5:**
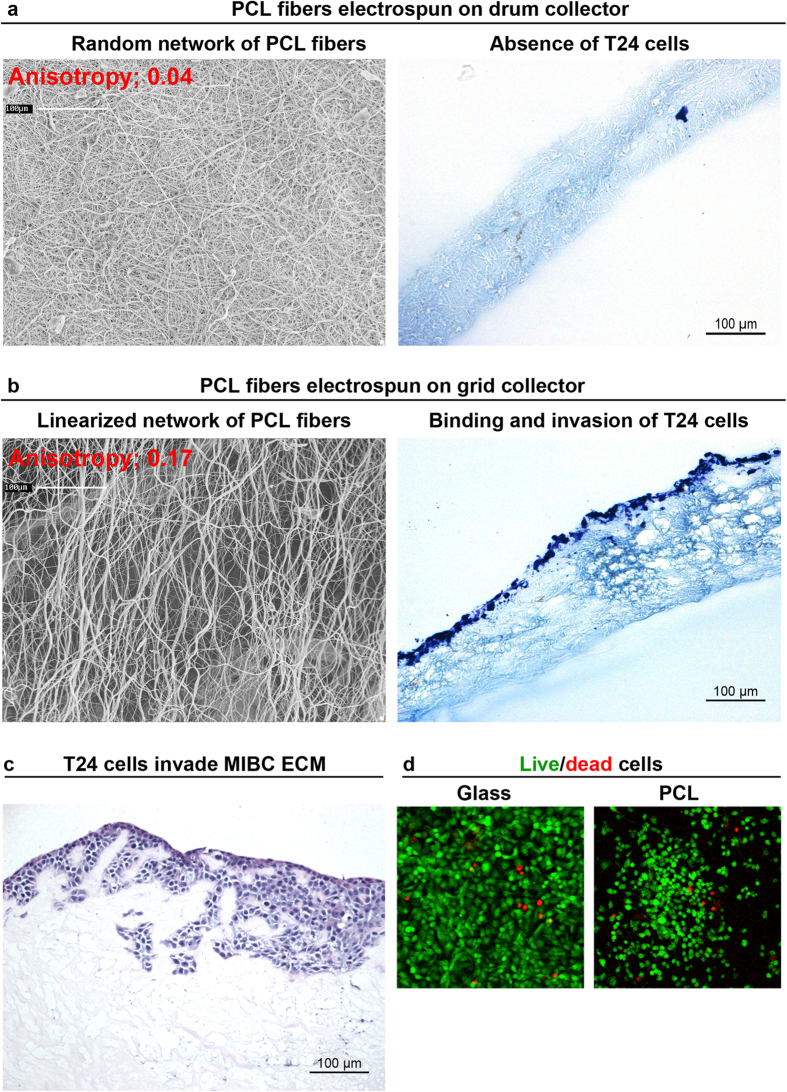
Linearized texture of synthetic scaffold allows cell invasion. Texture of electrospun scaffolds made of PCL fibers deposited on drum collector (**a**) and grid collector (**b**) were investigated by scanning electron microscopy (SEM) and assessed for the ability to support binding and invasion of metastatic T24 cells. The degree of fibril organization (anisotropy index) was estimated on the SEM micrographs by using ImageJ. Presence of T24 cells on electrospun PCL was analyzed 7 days after seeding; histology evaluation was performed on sections obtained from a transverse cut of the scaffold. Metastatic T24 cells were seeded onto MIBC-derived ECM, and 4 days after seeding 3 μm paraffin sections from each sample were used, and hematoxylin-eosin stained sections were histologically evaluated (**c**) one experiment representative of 3 independently performed on MIBC-ECM obtained from 3 different tumors is shown. Cell viability of cells seeded for 7 days on the synthetic scaffold was compared to T24 cells plated on glass-slide (green and red spots show live and dead cells, respectively) (**d**) For panels a,b and d one experiment representative of 3 independently performed is shown.

The first scaffold was obtained upon electrospun on rotating drum collector, resulting in the formation of synthetic fibers of 492 ± 18 nm (No. = 30 measurements) width, arranged in well-separated but intersecting fibers forming a random network with anisotropy of 0.04; on this scaffold metastatic T24 cancer cell lines did not bind (Fig. 5a).

The texture of the electrospun PCL was next changed in order to recapitulate the linearized geometry of MIBC-derived ECM. PCL was electrospun on grid collector, where the fibers are aligned in the center of the grid (Fig. 5b). With this geometry electrospun fibers were 701 ± 24 nm (No. = 30 measurements) width, and reached a degree of anisotropy of 0.17; T24 cells were bound on this scaffold with linearized texture and they successfully invaded the matrix 7 days after seeding (Fig. 5b), as observed on MIBC ECM (Fig. 5c).

Finally, PCL fibers did not modify cell viability that was comparable to that of cells cultivated in the absence of the scaffold itself (96% cell viability, Fig. 5d).

## Discussion

This study provided information regarding the ultrastructure and the geometry of a healthy human bladder ECM. Likewise, we compared these features with those observed in a set of urothelial MIBCs. Here we report novel evidence depicting differences and similarities in term of ECM ultrastructure between a non-tumoral and a MIBC human tissue, along with a number of detailed measurements of the geometry of the healthy lamina propria and muscularis propria. Second, the study showed that MIBC ECM is characterized by an increased degree of fibrils organization, in agreement with a recent report on colorectal carcinoma^22^. Third, using a synthetic scaffold this study showed that the linearized texture of fibrils is the main feature of the ECM associated with cell invasion. Findings of the present study are exploitable for the design of synthetic non-neoplastic bladder and MIBC scaffolds, and for assessing pathways associated with tumor invasion.

To the best of our current knowledge, data about the ultrastructure of human bladder mucosa and the muscularis propria, and the potential modifications of these tissues associated with a tumor invasion have not yet been reported. As for the healthy areas of bladder, different features of ECM were evident between the lamina propria and the muscularis propria, suggesting that for its specific structure the lamina propria acts as a capacitance layer, as it has previously been reported in the bovine bladder^36^.

The characteristics of the muscularis propria ECM outlined a fibrillar ultrastructure packed into intertwisted bundles. Those twists were quite close, as it would be expected for a relaxed bladder having performed the cystectomy on an empty organ.

Crests and clefts due to the presence of collagen bundles were evident in the ECM of the muscularis propria, as crests and clefts were resulting from the presence of a cytoskeleton (eg, α-smooth muscle actin and vimentin) preventing any structural collapse. The distance between bundles was in agreement with the size of smooth muscle cells in the detrusor layer of a bladder in the relaxed state^37^. Other features were indicative of a relaxed state of the analyzed bladders, including the round shape of “foot-like” depression in the basal lamina and the loose network observed in the lamina propria which was composed by crossed and intersected but not parallel fibrils.

In the context of tissue engineering the acquisition of an adequate knowledge regarding the spatial geometry of a potential synthetic bladder scaffold may be of paramount relevance. The maintenance of morphological and geometrical features of the ECM of the human bladder is certainly of potential relevance for the design of an innovative biosynthetic scaffold. Indeed, proper 3D geometry of the extracellular environment and an adequate space allocated for cell seeding are needed for cell engraftment and polarization which may eventually lead to vascularization and innervation, as it was recently reported with scaffolds made of synthetic polymers^12^,^13^,^14^.

As to the specific features of the MIBC ECM, current findings highlighted (i) a loss of tissue morphology; (ii) the linearization and degree of organization of fibrils of thin diameter; and, (iii) an increased vascularization (both veins and arteries). All these specific aspects emerge to be of relevant impact when designing a 3D model which may adequately resemble the spatial ultrastructure of a tissue niche either favoring or hindering the development and the progression of a tumor, especially with the aim of developing new types of therapy for which a three-dimensional context could represent a better and more effective field test that a simple two-dimensional reality.

To this regard, further studies should assess the pathways responsible for the formation of a compact ECM made of organized fibrils, since this tumor-related ECM did not appear solely as a consequence of a desmoplastic reaction but it is deeply reorganized, possibly impacting the bladder compliance of MIBC patients. As previously detailed, one of the major modifications caused by the MIBC is an increased tissue vascularization, which might be responsible to enhance oxygen-dependent cross-linking of collagen^38^, thus resulting in a stiffer and more compact ECM. In this context, MIBC-derived ECM might represent a useful scaffold for assessing vessel formation in neoplastic tissue and establishing permeability of drugs in the dense neoplastic matrix. Tissue architecture has recently been reported to be important also for predicting chemotherapy outcome^39^. Therefore, current findings could help to design synthetic scaffolds recapitulating tridimensional geometry of tumoral ECM, thus enhancing biological approximation of the native tumor microenvironment, thus allowing for preclinical approaches more sensitive in predicting clinical response to anticancer drugs.

Furthermore, this study reported the design of a functional synthetic scaffold resembling the linearized texture of MIBC ECM, supporting binding and invasion patterns of bladder cancer cells. Thereof, the scaffold with linearized texture is exploitable to assess cellular signaling pathways mediating invasion, with the subsequent potential assessment of novel therapeutic targets.

Overall, we therefore consider that these findings could be of critical relevance for a better understanding of the 3D structure related to the neoplastic infiltration of the tissue and for the potential design of novel synthetic scaffolds useful for both preclinical understanding of topical therapeutic approaches and for bladder engineering.

## Material and Methods

### Tissue specimens

Bladder transitional cell carcinoma specimens encompassing all tissue layers were obtained through the Unit of Pathology at the IRCCS Ospedale San Raffaele (Milan, Italy). A formal written consent was obtained by the local Institutional Review Board (Ethic Committee IRCCS Ospedale San Raffaele, protocol URBBAN, March 6 - 2014). Data collection and all experimental protocols were approved by the Ethic Committee IRCCS Ospedale San Raffaele, in accordance with the relevant guidelines and regulations outlined in the Declaration of Helsinki. All methods were carried out in accordance with the approved guidelines. All patients signed written informed consent agreeing to supply their own anonymous information for this and future studies.

Bladder specimens stored in OCT were histologically validated by means of hematoxylin-eosin slides. Based on histological evaluation, tissue layers from non-neoplastic bladder were separated into mucosa (urothelium and lamina propria) and muscularis propria. Tissue layers and MIBC were then split in two blocks and either fixed/embedded in formalin/paraffin or used unfixed for ECM isolation.

### Preparation of ECM

ECMs were isolated according to a protocol recently published and validated on the human colon^23^. A minor modification to the original protocol was introduced, as components of solutions A and B were merged together thus resulting in a solution composed of 86.8% 0.1x PBS, 5 mM EDTA, 10% DMSO, 1% Triton X-100, 1% protease inhibitor cocktail and 1% pen/strep. Briefly, 100 mg of tissue free of OCT and washed in PBS was placed in 10 ml of solution A + B and kept rotating overnight at 4 °C. Tissue was then washed with solution C and transferred into 10 ml of solution C (0.5 M NaCl in 0.1x PBS) and kept rotating for 8 hours at 4 °C. Tissue was then washed with solution D and transferred into 10 ml of solution D (10 mM sodium cholate hydrate in 0.1x PBS) and kept rotating overnight at room temperature. Tissue was then washed with Tris buffer (50 mM Tris-HCl pH 8, 2 mM MgCl_2_). ECM was then suspended in 1.5 ml of Tris buffer added of 100 U/ml Benzonase and incubated 8 hours at 37 °C in thermomixer. Finally, ECM was either fixed/embedded in formalin/paraffin for histo- and immunological analysis, or fixed in glutaraldehyde for scanning electron microscopy.

PBS and pen/strep were from Lonza (BioWhittaker). EDTA, DMSO, Tris-HCl, NaCl, sodium cholate hydrate, Triton X-100 and protease inhibitor cocktail were from Sigma Chemical Corp. MgCl_2_, was from BDH, and Benzonase was from Novagen (Merck Chemicals Ltd.).

### Histological and immunohistochemistry analysis

Cells and nuclei in the tissues and ECMs were evaluated by hematoxylin-eosin, cellular antigens (cytokeratin, vimentin and α-smooth muscle actin) and the presence of stromal component collagen IV by immunohistochemistry, as it has been previously reported^23^,^40^. Tissues and ECMs were fixed in 10% buffered formalin for 24–48 h at room temperature and then embedded in paraffin. Three μm paraffin sections from each sample were used and hematoxylin-eosin stained sections were histologically evaluated. For immunohistochemistry, sections were pretreated in a microwave oven, two cycles of 5 min each, at 780 W in 0.25 mM EDTA buffer, and then incubated for 2 hours with specific antibodies against human antigens and counterstained with hematoxylin. The reactions were revealed by non-biotin peroxidase detection system with 3,3′-diaminobenzidine free base as chromogen. Antibodies against human cytokeratin, vimentin, α-smooth muscle actin and type IV collagen (clone MNF116, V9, HHF35 and CIV22, respectively) were from Dako (Dako Italia S.p.A.).

### Scanning electron microscopy (SEM)

ECMs for SEM were prepared as detailed^23^. Briefly, ECM were fixed with 2.5% glutaraldehyde (25% solution, electron microscopy grade) in PBS for 24 hours, dehydrated in an ascending degree of ethanol (10–25–50–75–90–100%) and dried overnight in hexamethyldisilizane. ECMs were coated with gold-palladium after evaporation of hexamethyldisilizane and examined in a Leica S420 scanning electron microscopy. Fibrils width and degree of organization of fibrils (anisotropy) were estimated by using the function “measure” and the plug-in FibrilTool^41^ in the Image J software; 3 electron micrographs were evaluated for each donor. Anisotropy of ECM was measured on the entire area of electron micrographs, in order to avoid any bias due to the selection of areas excluding or over-representing certain fibers in the outputs. The degree of alignment of fibrils in pair-wised ECMs was estimated on images with the same magnification.

### Seeding of bladder cancer cells on MIBC-derived ECM

T24 cell line (CLS Cell Lines Service GmbH, Eppelheim, Germany) was seeded onto bladder tumor-derived ECM as previously reported^23^. Briefly, 3*10^6^ cells in 10 ml of culture medium (DMEM added of 10% FBS, 1% penicillin/streptomycin sulfate and 1% glutamine) were incubated overnight with MIBC-derived ECM. The day after, ECM was then transferred in 6 well/plate, with culture medium entirely replaced every 2–3 days. Histological analyses were performed on 3 μm paraffin sections stained with hematoxylin-eosin.

### Electrospinning, characterization and cleaning of PLC scaffolds

Poli-e-caprolactone (PCL) pellets (Mw = 80 kDa, Sigma-Aldrich) were dissolved at a 15 w/v ratio in a solvent solution 1: 1 (v/v) of Cloroform (CF, Carlo Erba) and N, N-Dimethylformamide (DMF, Carlo Erba) under constant stirring until the mixture was clear, viscous and homogenous in a 15% w/v rate. PCL solution was poured into a syringe capped with a 19 gauge blunt-tipped needle nozzle, then loaded into a syringe pump (KD Scientific, Holliston, MA). The needle tip was connected to a custom made high voltage supply (ΔV = 0–30 kV). The resulting PCL nanofibers were deposited on collectors with different topography, such as rotating drum or grid mesh grounded aluminum, placed at a fixed distance. In detail two different set of working parameters were used to electrospun PCL on: (i) Rotating drum collector, ΔV = 17.9 ± 0.5 kV, V = 3 ml/h, D = 18 cm; (ii) grid mesh collector, ΔV = 12.2 kV, V = 3 ml/h, D = 14 cm. To eliminate any possible chemical or biological contaminants, scaffolds were subjected to washing and sterilization protocols. Scaffolds were washed in demineralized water with several cycles of 10 minutes each, to help the detachment of the membrane from aluminum, to remove dust and any residues of the solvents used over the manufacturing process. Next, scaffolds were washed twice in 70% ethanol (30 minutes each wash), followed by 30 minutes wash in Milli-Q water. Lastly, the scaffolds were immersed in an antibacterial/antifungal solution for 15 minutes (repeated twice), dried under biological hood overnight and exposed to UV rays for 15 minutes (for each side).

Scanning electron microscopy was performed on gold-palladium coated scaffold and examined in a Leica S420 scanning electron microscopy.

### Cell seeding on synthetic scaffolds and histology

Scaffolds were pre-conditioned with culture medium (DMEM added of 10% FBS, 1% penicillin/streptomycin sulfate and 1% glutamine) for 24 hours. Metastatic bladder T24 cells were seeded on synthetic scaffolds at 5000 cells/cm^2^, for a total of 1 cm^2^ surface. Culture medium was replaced every 2 days. Seven days after seeding, scaffolds were fixed in 10% buffered formalin and snap-frozen. Eight μm section from OCT-embedded scaffolds were then stained with Toluidine-blue and mounted with Aqueous-mounting medium, to preserve cellular and stromal staining.

### Cell viability

Live/dead viability assay (Calcein 488 and Propidium Iodide 555, Invitrogen) was performed according to manufacturer instructions. Briefly, 7 days after culture the scaffold with the seeded cells was washed with PBS, spotted on glass slide and incubated for 45 minutes at room temperature with 2 μM Calcein-AM and 4 μM EthD-1 working solution. Following incubation 10 μl of the fresh LIVE/DEAD reagent solution were added on top of the scaffold. Live and dead cells were visualized under fluorescence microscope: live cells were calcein positive, showing in green (Ex 495/Em 635 nm) and dead cells were EthD-1 positive cells, showing in red (Ex 495/Em 635 nm).

### Statistic

Geometric features are represented as either mean ± standard error of the mean or range of values. Non parametric, paired and two-tailed statistic test was used and p value considered significant at <0.05.

## Additional Information

**How to cite this article**: Alfano, M. *et al*. Linearized texture of three-dimensional extracellular matrix is mandatory for bladder cancer cell invasion. *Sci. Rep.* **6**, 36128; doi: 10.1038/srep36128 (2016).

## Figures and Tables

**Table 1:** Characteristics of patients with transitional cell carcinoma.

#	Sex	Age at surgery	TURBT-THERAPY	Tumor staging	Location of tumor in the bladder
1	M	67	NO	T3BG3N1	Rear, left and right lateral wall, dome
2	M	78	NO	T3AG3N2	Rear and left lateral wall
3	M	76	NO	T3BG3N3	Dome
4	M	78	NO	T3B N0 M0	Rear, left and right lateral wall,
5	M	88	NO	T4AG3N2	Rear wall
6	M	67	NO	T3AG2N1	Rear and left lateral wall
7	M	78	NO	T3BG3N0	Rear, left and right lateral wall, base
8	M	71	NO	T3AG3N0	Rear wall
9	M	56	NO	T3BG3N1	Rear and left lateral wall, dome
10	M	75	NO	T3AG3N1	Left lateral wall

All patients were naïve for TURBT (Trans Urethral Resection Bladder Tumor) and therapy.
